# Balance and Mobility Impairment in Older Adults with Cardiovascular Disease Before and After Rehabilitation: A Narrative Review

**DOI:** 10.3390/jcm15176657

**Published:** 2026-08-28

**Authors:** Zhengyang Song, Sasha Douglas, Imran Khan Niazi, Yanxin Zhang, Jocelyne Benatar, Paul W. Marshall

**Affiliations:** 1 School of Exercise, Sport and Rehabilitation Sciences, Faculty of Science, University of Auckland, Auckland 1142, New Zealandsasha.douglas@auckland.ac.nz (S.D.); yanxin.zhang@auckland.ac.nz (Y.Z.); 2 Centre for Health and Rehabilitation Research, Faculty of Science, University of Auckland, Auckland 1142, New Zealand; 3 Centre for Chiropractic Research, New Zealand College of Chiropractic, Auckland 1060, New Zealand; imran.niazi@nzchiro.co.nz; 4 Greenlane Cardiovascular Service, Auckland City Hospital, Auckland 1142, New Zealand

**Keywords:** cardiovascular disease, cardiac rehabilitation, older adults, balance impairment, postural control

## Abstract

Balance impairment threatens mobility and independence in older adults with cardiovascular disease, yet cardiac rehabilitation (CR) has traditionally prioritised aerobic capacity and cardiovascular outcomes. This narrative review examines the mechanisms and assessment of balance impairment, balance recovery within CR, and implications for clinical practice. Balance impairment reflects interactions among musculoskeletal, sensory, cognitive–motor, and cardiovascular constraints that affect different domains of postural control to varying extents. Standardised clinical measures do not capture these domains equally, and single scores or completion times can obscure the deficits underlying poor performance. Instrumented and wearable technologies extend clinical assessment by quantifying postural sway and gait, helping to distinguish broader mobility gains from recovery within specific balance domains. To translate this distinction into practice, this review recommends individualised, balance-focused CR, with assessment guiding task-specific training and virtual reality or exergaming providing graded practice and performance feedback to promote mobility and independence.

## 1. Introduction

Cardiovascular disease (CVD) remains the leading cause of death worldwide, with global deaths reaching 19.2 million in 2023 [1]. Population ageing and the age-related rise in disease prevalence are increasing the number of older adults living with CVD [2,3,4,5]. Multimorbidity and frailty are common in this population and increase vulnerability to functional decline [6,7,8,9]. Although advances in cardiovascular prevention and treatment have improved survival, many older adults with CVD continue to experience limitations in daily functioning [10,11,12,13]. These limitations compromise independence, underscoring functional recovery as a central care goal for older adults with CVD [4,14,15].

Older adults with CVD commonly experience reduced strength, slower gait, and impaired balance, which can limit their ability to rise from a chair, walk safely, and perform routine household activities [7,13]. Balance and mobility limitations are particularly important because they threaten independence, increase fall risk, and, in the case of slower gait, are associated with hospitalisation [7,16,17]. Balance and mobility should be recognised as central targets of functional recovery in older adults with CVD, rather than as secondary concerns.

Exercise-based cardiac rehabilitation (CR) has traditionally centred on aerobic training, with established benefits for exercise capacity and cardiovascular outcomes [18,19]. However, conventional programmes may not adequately address strength, mobility, and balance in older adults, potentially limiting broader functional recovery [11]. Among these functional domains, balance has received limited attention within CR [4,19]. Targeted strategies, including combined resistance and balance training and robot-assisted balance exercise, have yielded functional gains but no consistent advantage over conventional approaches [11,20]. Determining how to assess and address balance impairment within CR is a key clinical and research priority for older adults with CVD.

This narrative review provides a critical evaluation of the evidence for balance impairment and recovery in older adults with CVD and examines how balance-focused assessment and training might be integrated into CR. The first section provides a synthesis of evidence on functional impairments and the clinical significance of balance and mobility limitations. Subsequent sections evaluate the mechanisms underlying balance impairment and the evidence for conventional CR, targeted balance and multicomponent training, and technology-assisted approaches, including virtual reality (VR) and exergaming. Finally, requirements for safe, individualised implementation of balance-focused training within CR are presented, together with priorities for future research.

### Literature Search

This article is a narrative review and is reported in accordance with the Scale for the Assessment of Narrative Review Articles (SANRA) [21]. PubMed, Scopus, and Web of Science were searched from January 2000 to July 2026, supplemented by citation tracking of retrieved articles and targeted searching of relevant clinical practice guidelines. Search terms combined concepts relating to the population (older adult*, aged, elderly, cardiovascular disease, heart failure, coronary artery disease, cardiac surgery), the construct of interest (balance, postural control, postural stability, gait, mobility, falls), and the intervention context (cardiac rehabilitation, exercise training, balance training, virtual reality, exergaming). English-language primary studies, systematic reviews, and meta-analyses in adult populations were eligible for inclusion. Consistent with the aims of a narrative review, articles were selected purposively to provide conceptual coverage across mechanisms, assessment, and intervention, rather than through exhaustive protocol-driven screening; accordingly, no formal risk-of-bias appraisal, quality scoring, or quantitative pooling was undertaken, and the synthesis presented is interpretive rather than statistical. Where direct evidence in older adults with cardiovascular disease was limited, evidence from healthy older adults or from other clinical populations has been drawn upon and is identified as extrapolated at the point of use.

## 2. Functional Impairments in Older Adults with Cardiovascular Disease

Ageing is accompanied by increased myocardial stiffness and impaired coronary vasodilatory function, reducing cardiovascular reserve and increasing vulnerability to CVD. These changes interact with the underlying disease processes, increasing vulnerability to functional decline [22,23]. Older adults with CVD commonly experience multidimensional functional impairments, including reduced mobility, muscle weakness, and impaired balance [8,13]. These impairments may manifest as slower gait, difficulty rising from a chair, and reduced capacity for daily activities, thereby limiting independence [7,13,24]. The 6-min walk test, Timed Up and Go test (TUG), and grip strength provide complementary information on exercise capacity, mobility, and muscular strength in older adults with CVD [8,13,25].

### 2.1. Gait and Mobility

Gait speed is a clinically meaningful indicator of mobility and functional vulnerability [17,26,27,28]. In patients with stable CVD, each 1 km·h^−1^ increase in walking speed was associated with lower one-year hazards of all-cause and cardiovascular hospitalisation, with hazard ratios of 0.83 and 0.84, respectively [17]. In community-dwelling older adults, each 0.1 m·s^−1^ faster gait speed was associated with a mortality hazard ratio of 0.88 [27].

Walking requires coordinated cardiopulmonary, neurological, and musculoskeletal function, making gait speed a broader indicator of functional mobility rather than an isolated measure of walking performance [27]. Older adults with heart failure demonstrate substantial mobility impairment, with frailty independently associated with poorer TUG performance and gait speed approximately 35–40% below population norms [13,29]. Because gait speed may improve with CR and short-term changes can reflect clinically meaningful functional recovery, repeated measurement may help track mobility-related change during rehabilitation [7,30].

### 2.2. Muscular Weakness

**Figure 1:**
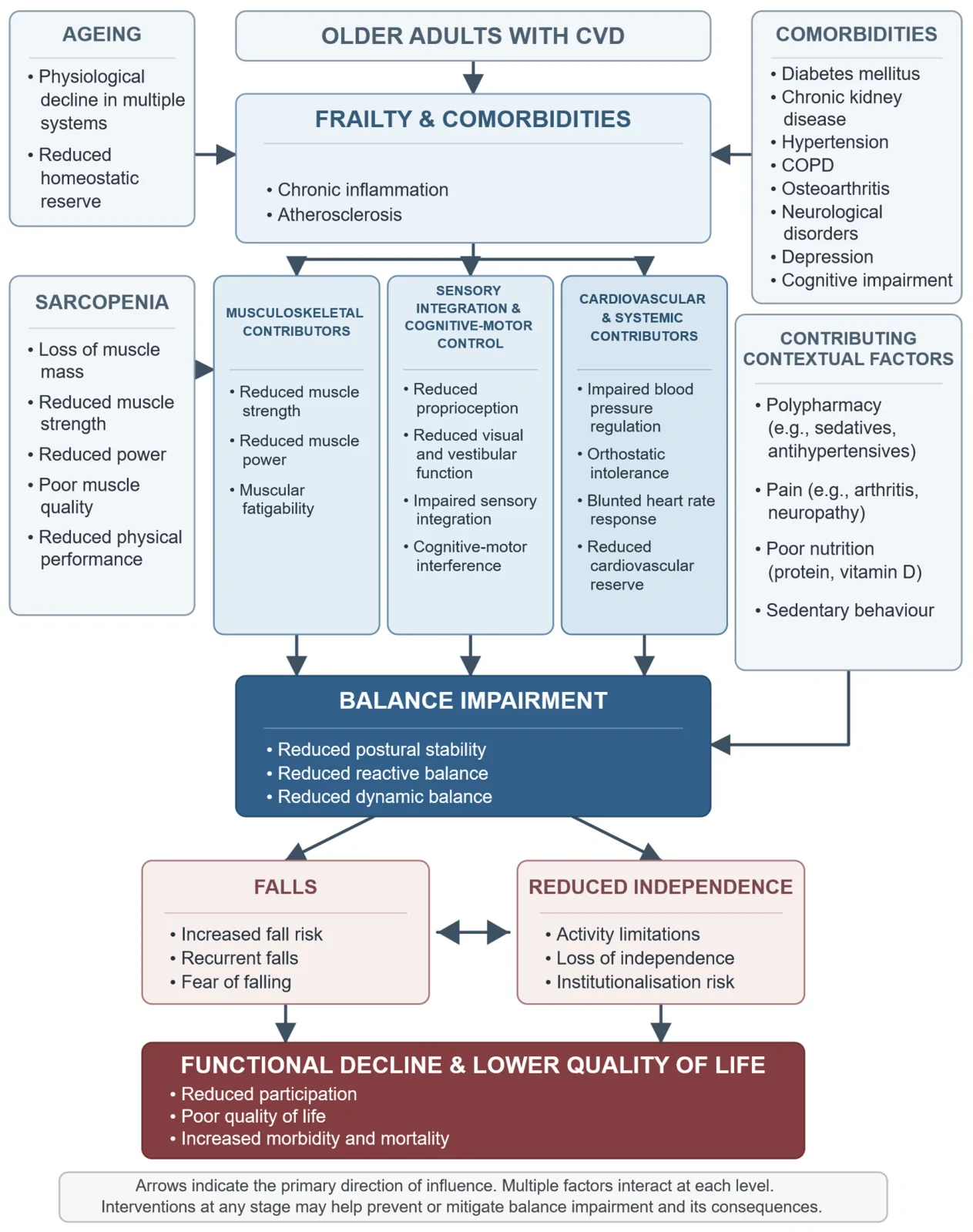
Conceptual framework of factors contributing to balance impairment in older adults with cardiovascular disease. Upstream age-related, clinical, and contextual factors may contribute to balance impairment through multiple pathways. The principal mechanisms are organised into three domains: musculoskeletal contributors, sensory integration and cognitive–motor control, and cardiovascular and systemic contributors. Balance impairment may manifest as reduced postural stability, reactive balance, and dynamic balance, with downstream consequences including falls, reduced independence, functional decline, and lower quality of life. Abbreviations: COPD, chronic obstructive pulmonary disease; CVD, cardiovascular disease.

In older adults with CVD, muscle weakness is associated with frailty, impaired mobility, and adverse clinical outcomes [31,32]. Low grip strength is a criterion of the Fried frailty phenotype and was lower in frail than non-frail CR participants [8,33]. In an individual-participant data meta-analysis of seven studies involving 23,480 patients with cardiac disorders, each 5-kg higher grip strength was associated with lower odds of cardiac death, all-cause death, and hospital admission for heart failure, with odds ratios of 0.84, 0.87, and 0.88, respectively [34]. Lower-limb weakness is also evident in heart failure, with reduced quadriceps strength relative to healthy controls [35] and lower knee extensor strength independently associated with poorer TUG performance in older patients [13]. These findings support including grip and lower-limb strength in CR assessment, particularly when muscular weakness may contribute to mobility limitations. The broader factors contributing to balance impairment in older adults with CVD are summarised in Figure 1, which provides a conceptual framework for the three mechanistic domains discussed below.

### 2.3. Balance Impairment in Older Adults with Cardiovascular Disease

Balance is a multidimensional function that depends on musculoskeletal capacity, sensory integration, and neural control [36]. Balance impairment reflects multiple interacting physiological constraints rather than a uniform deficit [35,37,38]. In older adults with CVD, age-related declines in musculoskeletal capacity, sensory reliability, and cognitive–motor control may interact with CVD-related peripheral muscle dysfunction, exercise intolerance, and haemodynamic instability [39,40,41]. Consequently, balance impairment in older adults with CVD should be characterised across specific domains of postural control rather than reduced to a single test score.

#### 2.3.1. Clinical Domains and Assessment of Balance Impairment

Postural control is a complex motor skill arising from the interaction of multiple sensorimotor processes, including anticipatory postural control, reactive postural control, sensory integration, and dynamic stability [37,42]. However, these components are not consistently represented across standardised balance measures. In a scoping review of 66 standardised balance measures, 34 assessed three or fewer components of balance, with reactive postural control and cognitive influences evaluated in 23% and 17% of measures, respectively [42]. The TUG provides a brief measure of functional mobility, dual-task variants incorporate cognitive–motor demand, and the Mini-Balance Evaluation Systems Test (Mini-BESTest) assesses a broader range of postural control components [43,44,45]. These measures should be regarded as complementary rather than interchangeable when characterising balance impairment in older adults with CVD.

Evidence from chronic heart failure suggests that balance impairment is not uniform across postural control components. Patients with chronic heart failure had lower Mini-BESTest scores than healthy controls, with mean scores of 22.1 and 26.6, respectively, and significant subscale differences confined to reactive postural control and gait stability, while anticipatory postural adjustments and sensory orientation did not differ between groups [35]. Although clinically informative, this domain-specific pattern may indicate potential heterogeneity in balance impairment, but its generalisability to older adults across the broader spectrum of CVD is limited by the small cohort and mean age of approximately 59 years. Larger studies across a broader range of cardiovascular conditions are needed to determine whether similar domain-specific patterns are present.

A further limitation of standardised clinical balance measures is that they summarise balance through ordinal scores or completion times, which capture the outcome of a task but not the postural control process that produced it [46]. Instrumented assessment provides continuous quantitative information about that process. Force-plate posturography quantifies centre-of-pressure excursion, sway area, and sway velocity, and body-worn inertial sensors provide measures of postural sway during standing and of spatiotemporal parameters during walking [46]. These centre-of-pressure measures have shown good-to-excellent test–retest reliability in older cardiac rehabilitation patients [47]. Evidence in healthy older adults suggests that these systems can help distinguish aspects of postural control that clinical tests aggregate, for example by contrasting sway under eyes-open and eyes-closed conditions or on firm and compliant surfaces to probe sensory reweighting [48]. In healthy populations, smartphone-based systems have brought this measurement out of the laboratory, with a validated application deriving postural stability, walking speed, and step measures from a single waist-worn sensor across standing and walking tasks, and showing sensitivity to age and agreement with laboratory and clinical measures [49,50,51]. These approaches align with the domain-specific assessment this review advocates and with the practical constraints of cardiac rehabilitation, where brief testing with minimal setup is needed. Because validity varies across wearable sensor-derived outputs, further work should establish which measures can identify specific balance deficits and detect change in older adults with CVD [52].

#### 2.3.2. Musculoskeletal Contributors

Musculoskeletal capacity supports postural control by generating the force needed to maintain alignment and rapidly execute corrective responses. Age-related reductions in lower-extremity strength and power may limit the speed and magnitude of postural corrections. However, muscle weakness should not be treated as a direct surrogate for balance impairment. A systematic review and meta-analysis of 37 studies in healthy populations found generally weak associations between lower-extremity strength or power and balance, with a pooled correlation of r = 0.35 between maximal strength and dynamic steady-state balance in adults aged 65 years or older [53]. Muscular capacity is best understood as one constraint on postural control that interacts with sensory and neural processes, rather than as a sufficient explanation for balance performance.

The modest association between maximal strength and balance may partly arise because musculoskeletal contributions to postural control extend beyond maximal force-generating capacity and include muscular endurance and fatigability, which are not captured by conventional measures of maximal strength. In heart failure, impaired skeletal muscle perfusion, oxygen extraction, and mitochondrial function may accelerate exertional fatigue and reduce the muscular reserve available during prolonged standing and walking [39,54]. A systematic review of seven small studies, conducted largely in healthy community-dwelling older adults, found that six reported significant adverse effects of lower-extremity or trunk muscle fatigue on balance or functional performance, including postural stability, functional reach, sit-to-stand performance, and gait stability [55]. Although these studies did not include cardiac populations, the findings suggest that muscle fatigue may impair postural control in ways not captured by maximal strength.

#### 2.3.3. Sensory Integration and Cognitive–Motor Control

Postural control requires visual, vestibular, and proprioceptive information to be integrated and weighted according to the reliability of each source [56,57]. When one source becomes unreliable or unavailable, the nervous system must rapidly reweight sensory inputs towards more reliable sources to preserve orientation and stability [57]. Age-related declines in proprioceptive acuity and central integration may limit adaptive sensory reweighting [58,59]. Balance may remain adequate under predictable conditions yet deteriorate when visual input is reduced, the support surface becomes unstable, or competing sensory information must be resolved.

Postural control also draws on attentional resources, particularly when balance or gait must be maintained during a concurrent task [60,61]. With ageing, balance and gait may place greater demands on attention, leaving less reserve for a concurrent task [62]. A meta-analysis of 66 dual-task gait studies found that concurrent cognitive tasks reduced gait speed in healthy participants, with mean reductions ranging from 0.02 m·s^−1^ for discrimination and decision-making tasks to 0.12 m·s^−1^ for verbal fluency tasks [63]. This variation suggests that cognitive–motor interference is task specific rather than a uniform effect on mobility. Whether this mechanism is amplified in older adults with CVD remains uncertain because few studies have quantified dual-task costs in this population.

#### 2.3.4. Cardiovascular and Systemic Contributors

Standing imposes both postural and haemodynamic demands, with approximately 500–1000 mL of blood shifting from the central circulation to the lower extremities and splanchnic vascular bed, thereby reducing venous return [40]. Autonomic increases in heart rate and vascular tone normally preserve cardiac output and cerebral perfusion, whereas an inadequate compensatory response may result in orthostatic hypotension and transient cerebral hypoperfusion [40,64].

A meta-analysis of 25 studies found that older adults with orthostatic hypotension had 39% higher odds of falling within 12 months than those without orthostatic hypotension [65]. However, the pooled estimate was unadjusted, most included studies were observational, and heterogeneity in orthostatic hypotension definitions, measurement protocols, medication exposure, and comorbidity limits causal interpretation. Orthostatic hypotension should therefore be viewed as a marker of cardiovascular vulnerability associated with falls rather than as an established independent cause.

Falls are multifactorial and not specific to impaired postural control. Associations with cardiovascular instability alone cannot establish whether or how balance is affected. Cardiovascular conditions may affect postural control through transient cerebral hypoperfusion, exertional fatigue, medication-related haemodynamic effects, and reduced physiological reserve during postural challenges [39,40,65]. Studies combining haemodynamic monitoring with domain-specific balance tasks are needed to determine whether and under what conditions these systemic constraints impair postural control.

## 3. Balance and Mobility Recovery in Cardiac Rehabilitation

A central clinical question is whether CR improves balance and mobility beyond its established effects on general exercise capacity [19,66]. Conventional CR may enhance walking endurance and functional mobility, but such changes should not be assumed to represent recovery across all domains of postural control. In this review, we distinguish balance-specific outcomes from broader functional outcomes, including measures of mobility, physical performance, and exercise capacity. TUG and gait speed are treated primarily as mobility measures, the SPPB as a composite measure of physical performance that includes a standing-balance component, and the 6MWT as a submaximal measure of exercise capacity [27,44,67,68]. Improvements in these measures are therefore not interpreted in isolation as evidence of balance-specific recovery. Conventional CR, targeted balance and multicomponent training, and VR or exergaming differ in therapeutic focus and task demands and should not be treated as equivalent approaches. Table 1 summarises the clinical evidence across these approaches.

Taken together, the studies summarised in Table 1 suggest more consistent improvements in mobility, physical performance, and exercise capacity than in balance-specific outcomes. Although favourable effects of balance or multicomponent training have been reported, improvements vary across balance domains and are not consistently greater than those observed with usual rehabilitation. Interpretation of the evidence is limited by heterogeneity in cardiovascular populations, intervention content and dose, study design and sample size, attrition and adherence, outcome selection, and the limited assessment of longer-term retention.

### 3.1. Functional Outcomes Following Conventional Cardiac Rehabilitation

Conventional CR may improve mobility and lower-extremity function without producing comparable gains in balance. In adults aged 65 years or older completing phase II CR, the total Short Physical Performance Battery (SPPB), a composite measure comprising standing balance, gait speed, and repeated chair-stand performance, increased by 0.8 points, below the one-point threshold generally considered clinically meaningful [67]. This modest overall change was driven by improvements in gait speed and chair-stand performance, while the balance component remained unchanged. Participants with lower baseline physical function showed a larger 1.6-point increase in total SPPB, suggesting greater scope for improvement among more impaired patients, although the overall pattern still primarily reflected gains in mobility and lower-extremity function rather than balance recovery.

An unchanged balance component does not establish that complementary resistance and balance training will add functional benefit to conventional CR. In a randomised trial of 116 older adults after valve surgery or intervention, usual CR and additional resistance-and-balance training produced similar improvements in walking capacity, physical performance, and gait speed, with no significant between-group differences [76]. The added benefit of complementary training may be difficult to detect when usual CR already improves overall function and the selected outcomes primarily capture global performance rather than domain-specific balance.

### 3.2. Targeted Balance and Multicomponent Training

Among 349 older adults hospitalised with acute decompensated heart failure, an individualised and progressive programme targeting balance, strength, mobility, and endurance improved SPPB score by 1.5 points and 6-min walk distance by 34 m relative to attention control, with both changes exceeding accepted thresholds for clinical importance [74]. Almost all participants were frail or prefrail at baseline, which may have provided greater scope for improvement across multiple functional domains. However, because the four domains were trained concurrently and SPPB is a composite measure, the independent contribution of balance training cannot be determined.

An exploratory randomised trial more directly tested balance-focused training by replacing 20 min of conventional warm-up with stability and coordination exercises while keeping the total duration of CR unchanged. Among 26 older adults with CVD, targeted training reduced TUG completion time by 0.98 s and Balance Error Scoring System (BESS) score by 6.7 points, with no corresponding changes in the control group, whereas Functional Reach did not improve [72]. Because the BESS captures static stability and sensory integration, whereas Functional Reach reflects functional stability limits and anticipatory postural control [42], the contrasting responses across these measures suggest that improvements were confined to outcomes more closely aligned with the postural demands emphasised during training rather than extending uniformly across balance domains. However, 14 of the 40 randomised participants withdrew, intervention adherence was 70%, and part of the BESS improvement was lost four weeks after training, limiting confidence in the magnitude and durability of the effect.

These trials show that balance-focused training varies substantially in content, dose, and clinical context. In 173 adults aged 75 years or older following coronary artery bypass surgery, adding resistance and balance training five days per week to three weeks of inpatient CR produced an approximately 25 m greater improvement in 6-min walk distance and a 1.2 s greater reduction in TUG time than usual rehabilitation alone [70]. By contrast, trials in older adults following acute coronary syndrome or valve surgery found no additional improvement in walking capacity, SPPB score, gait speed, or leg strength when complementary resistance and balance training was delivered three times weekly alongside usual rehabilitation [11,76]. Although differences in patient populations and usual-care programmes preclude attributing the contrasting findings to training dose alone and limit their generalisability across cardiovascular populations, the pattern suggests that incremental benefit may depend on the specificity, progression, and dose of training relative to baseline deficits. Multidomain assessment can align balance training and outcome selection with the postural control deficits identified at baseline.

### 3.3. Virtual Reality and Exergaming for Balance and Mobility Training

The evidence base for VR and exergaming in balance rehabilitation is best characterised as emerging rather than established. Most available trials have been conducted in community-dwelling older adults or in neurological populations, and cardiovascular-specific evidence remains confined to small feasibility studies and a limited number of trials conducted within CR. The findings summarised below should therefore be read as indicating plausible mechanisms and preliminary signals of benefit rather than demonstrated efficacy in older adults with CVD.

The clinical value of VR and exergaming lies less in technological novelty than in the capacity to deliver purpose-built balance and mobility training. Across 15 randomised trials involving 870 community-dwelling older adults, purpose-built exergames produced a moderate improvement in balance outcomes, with a standardised mean difference of 0.75 [78]. A separate meta-analysis of immersive VR found improvements in standing balance and Berg Balance Scale scores, but not gait, functional mobility, or fear of falling [79]. In women aged 75 years or older, VR-assisted dual-task training and the conventional Otago Exercise Programme produced distinct outcome profiles rather than consistent superiority of either intervention. In the VR-assisted dual-task group, closed-eye single-leg stance increased from 1.52 to 2.33 s and post-training dual-task measures generally favoured VR, whereas standard TUG and Berg Balance Scale outcomes favoured the conventional Otago Exercise Programme [80]. Together, these findings suggest that the effects of VR are task specific and depend on exercise prescription rather than immersion alone.

Individualised training requires both an appropriate starting prescription and continued adjustment as performance changes. Clinically oriented systems have used functional and biomechanical assessment to guide initial task difficulty and then adapt task demands and feedback during training [81]. Together, these features link assessment-guided prescription with performance-based progression and real-time movement feedback.

Individualisation is clinically useful only when the resulting system remains usable for patients [82,83] and manageable for clinicians. Preliminary studies across neurological and CR settings have reported acceptable usability and favourable patient and clinician responses to clinically oriented VR systems [81,84,85]. However, positive overall ratings have coexisted with practical problems involving task difficulty, interface comprehension, and movement execution. Lower ratings for difficulty appropriateness suggest that positive overall usability does not ensure that task demands are well matched to patient capacity [85]. In CR, usability must also be considered alongside physiological monitoring and safe workload regulation. In a study involving 10 patients with heart failure, participants reported a mean System Usability Scale score of 71.5 and very high enjoyment, yet 76 of 136 observed events were classified as negative, most involving confusion [84]. Clinical use in CR requires systems that combine adaptive progression and simple operation with cardiovascular monitoring and clinician oversight.

VR and exergaming have shown balance benefits in older adults, adaptive delivery in post-stroke rehabilitation, and preliminary feasibility in CR [78,79,81,84], with a randomised trial involving older adults with CVD reporting that video-game balance training using a balance exercise assist robot and conventional resistance training yielded comparable improvements in TUG and SPPB [20]. A meta-analysis of 20 CR trials found that VR-based interventions improved 6-min walk distance by 34.9 m and quality of life, but did not evaluate balance-specific outcomes [86]. Accordingly, VR and exergaming are best viewed as adjunctive platforms for delivering individualised balance and mobility training within CR. VR and exergaming should be regarded as promising but not yet established components of balance-focused CR, and their routine adoption cannot presently be supported by cardiovascular-specific trial data.

## 4. Clinical Implications for Balance-Focused Cardiac Rehabilitation

The clinical integration of balance-focused training is challenged by the heterogeneous and domain-specific nature of balance impairment in older adults with CVD. The choice of balance and mobility measures should reflect the patient’s functional presentation, clinical context, and the practical capacity of the CR programme [87,88]. Conventional measures of exercise capacity and global physical function remain clinically informative, but they do not identify which domains of postural control are impaired [42,68]. Brief measures such as gait speed or the TUG may provide an initial indication of mobility limitation, whereas more targeted assessment may be required when instability is task specific, emerges under cognitive or sensory challenge, or involves reactive balance [35,42]. These measures are complementary rather than interchangeable, and a fixed assessment battery is unlikely to suit every patient or CR programme. The purpose of assessment is not simply to classify balance impairment, but to identify the functional limitations that subsequent training is intended to address.

For many older adults participating in CR, maintaining or regaining independence in everyday mobility is an important goal. Assessment findings should guide exercise prescription in line with each patient’s goals and priorities [87,89]. An older patient with CVD may value walking independently outdoors and remaining stable during turns or distractions more than improving an isolated balance score. Achieving such goals may require progressively challenging, task-specific practice under complex or distracting conditions, with VR-assisted or exergame-based training offering one adjunctive means of delivering this practice with performance feedback.

Safe balance-focused training requires the level of supervision and clinical input to reflect each patient’s functional complexity and cardiovascular risk [87,88]. Exercise professionals can guide task selection and progression while monitoring movement quality and physiological tolerance. Physiotherapy input may be particularly relevant for recurrent falls, gait abnormalities, marked instability, or assistive-device needs, whereas medical or nursing review may be required when dizziness, orthostatic symptoms, medication effects, or haemodynamic instability complicate training. The level of direct observation required should also inform the choice of delivery model. Hybrid or home-based delivery may expand access, but reduced observation increases the importance of clear monitoring procedures and periodic reassessment [90,91]. VR-assisted or exergame-based training may support selected balance tasks but should remain embedded within clinician-led progression and safety oversight.

Safety planning should begin before balance training is prescribed. A pre-training review should establish resting and standing blood pressure, any history of dizziness, presyncope, syncope, or unexplained falls, and the current medication profile, since diuretics, vasodilators, alpha-blockers, and other antihypertensive agents increase the likelihood of orthostatic intolerance during standing tasks [40,64,65]. Beta-blockade blunts the heart rate response to exertion, so heart rate alone is an unreliable guide to training intensity in many cardiac patients, and symptom reporting and rating of perceived exertion should be used alongside it. Training should be paused for dizziness, lightheadedness, chest discomfort, disproportionate breathlessness, palpitations, or a symptomatic fall in blood pressure, and resumed only after clinical review. In the early period following sternotomy, sternal precautions restrict the use of upper-limb support during balance recovery and should shape both task selection and the form of physical assistance provided.

The training environment requires equal attention. Standing balance tasks should be performed within immediate reach of a stable support, on a clear non-slip surface, in footwear appropriate to the task, and with a supervising clinician positioned to provide contact assistance during high-challenge conditions such as a reduced base of support, altered surface compliance, or eyes-closed and dual-task variants. Technology-assisted delivery introduces further considerations. Head-mounted immersive systems restrict peripheral vision and awareness of the immediate environment, can provoke visual-vestibular conflict with associated nausea and disorientation, and may leave transient unsteadiness on headset removal; initial familiarisation is therefore best conducted seated or with support, exposures kept short, and standing immersive tasks reserved for supervised sessions [92,93,94,95,96]. Cabled systems and floor-mounted sensors introduce trip hazards that should be cleared from the working area. Where hybrid or home-based delivery is used, the reduction in direct observation should be matched by a more conservative prescription, restricting unsupervised practice to tasks that can be performed safely with a fixed support available, together with explicit stopping instructions and a defined route to clinical review [90,91].

### Translating Current Evidence into Clinical Practice

**Figure 2:**
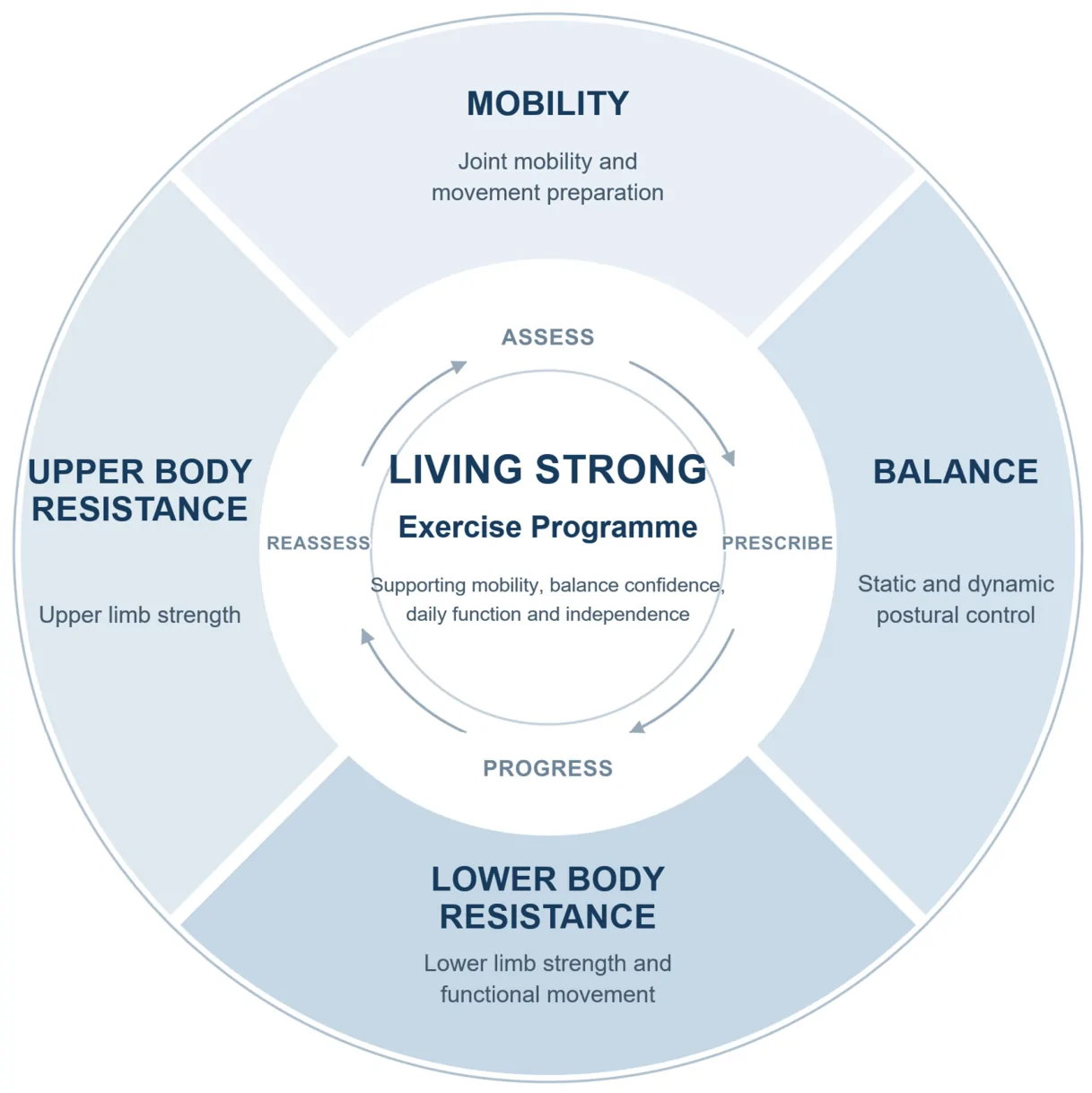
Key components and implementation process of the Living Strong exercise programme within cardiac rehabilitation, including integration into daily routines through weekly planning and habit cues.

The evidence reviewed has practical implications for programme development within CR. Following implementation of a previously developed home-based resistance exercise programme for cardiac rehabilitation [18], nursing staff identified that a proportion of older and frailer patients remained unable or unwilling to complete the prescribed exercises because of limitations in balance, mobility and confidence. Rather than simply reducing exercise volume or intensity, a modified programme was developed using the mechanistic and clinical principles outlined throughout this review. Indirect evidence from falls prevention [97], frailty rehabilitation [98], multicomponent exercise training and balance-specific interventions informed selection of exercises emphasising mobility, postural control, lower-limb strength and progressive functional challenge. The resulting Living Strong programme was subsequently refined through iterative feedback from cardiac rehabilitation nurses and patients to maximise usability, safety and feasibility within home-based and outpatient cardiac rehabilitation settings. Although developed as a clinical service initiative rather than a formal intervention trial, the programme illustrates how current evidence can be translated into an accessible balance-focused rehabilitation resource for older adults with cardiovascular disease. The key components and implementation process of the Living Strong programme are summarised in Figure 2.

The complete Living Strong programme, including exercise instructions, progression strategies, and safety guidance, is provided as Supplementary Material to illustrate one example of how balance-focused exercise prescription can be incorporated within cardiac rehabilitation. The programme is not intended as a prescriptive protocol but as an example of how current evidence may be translated into an individualised, progressive exercise resource suitable for adaptation to local services and patient needs.

## 5. Gaps in Evidence and Future Research Directions

Post-intervention follow-up is limited in CR trials reporting balance and mobility gains, leaving their durability uncertain. Hashimoto et al. [99] reported improvements in gait speed, TUG performance, and SPPB scores after four months of outpatient CR incorporating robot-assisted balance training, but the absence of post-intervention follow-up prevented assessment of whether these gains persisted after training. Segev et al. [72] found that BESS improvement had partially diminished four weeks after training ended, suggesting that balance gains may not be fully sustained. Future trials should reassess the same domain-specific outcomes after training to determine whether gains persist.

Such reassessment requires well-characterised measurement tools. A further priority is therefore to establish the measurement properties of instrumented and wearable balance assessment in older adults with cardiovascular disease. Sensor-derived sway and gait measures can quantify aspects of postural control that standardised clinical balance measures aggregate, but their reliability, responsiveness, and agreement with established measures remain incompletely characterised in this population [46,47,52].

**Figure 3:**
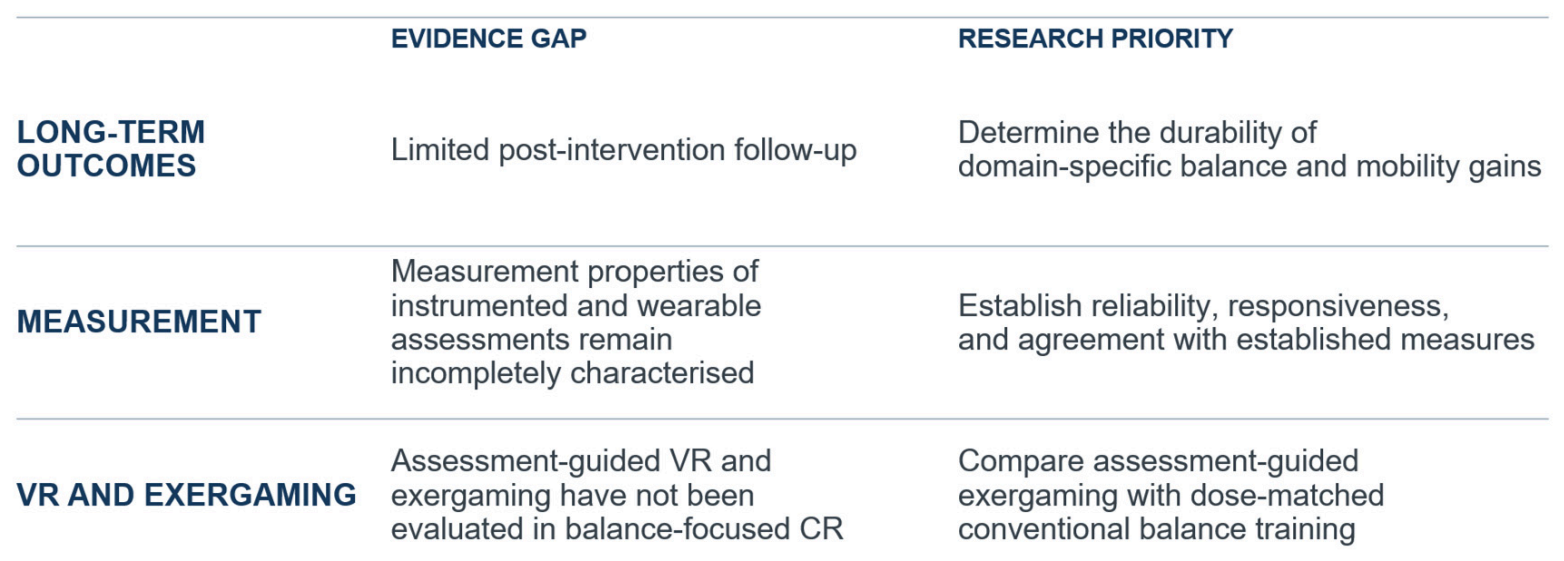
Evidence gaps and research priorities for balance-focused cardiac rehabilitation in older adults with cardiovascular disease. Priorities include determining the durability of domain-specific balance and mobility gains, establishing the reliability and responsiveness of instrumented and wearable assessments and their agreement with established measures, and comparing assessment-guided exergaming with dose-matched conventional balance training. Abbreviations: CR, cardiac rehabilitation; VR, virtual reality.

Another priority is to test whether VR and exergaming can support individualised progression and repeated performance assessment under clinician oversight within balance-focused CR. Clinically oriented systems have already used performance data to adapt task difficulty and provide individualised feedback during training [81]. In cardiovascular populations, physiological monitoring and clinician-set intensity targets would also be required, as incorporated into a preliminary exergaming prototype for patients with heart failure [84]. These capabilities have not yet been combined and evaluated in balance-focused CR for older adults with CVD. Future trials should compare assessment-guided exergaming with dose-matched conventional balance training to determine whether exergaming improves domain-specific balance and mobility, supports sustained participation, or enhances movement quality. The key evidence gaps and corresponding research priorities are summarised in Figure 3.

## 6. Conclusions

Balance impairment in older adults with CVD is multidimensional and is not fully captured by measures of aerobic capacity or global physical function. Conventional CR can improve walking and overall physical function, but these gains do not consistently extend across all domains of postural control. Targeted, multicomponent, and technology-assisted approaches have shown selective benefits, but no single strategy has demonstrated consistent superiority. Balance recovery is task-specific and depends on the functional deficits targeted during training.

CR for older adults with CVD should assess balance and mobility alongside exercise capacity. Assessment findings should guide the selection and progression of balance tasks rather than lead to a standardised programme. VR and exergaming should complement rather than replace conventional rehabilitation by providing graded balance tasks and performance feedback. Research should now establish whether assessment-guided balance training produces durable, domain-specific gains and whether VR and exergaming offer additional benefit over dose-matched conventional training.

As cardiac rehabilitation continues to evolve towards more individualised and function-focused models of care, balance assessment and training should be increasingly integrated into rehabilitation rather than an adjunct to aerobic exercise. The challenge is no longer determining whether balance matters in older adults with cardiovascular disease, but translating the growing evidence base into practical, evidence-informed programmes that support functional recovery, independence, and healthy ageing.

## Figures and Tables

**Table 1:** Overview of clinical studies reporting balance and functional outcomes in cardiac rehabilitation and cardiovascular disease populations.

Year and Study	Population and Design	Intervention	Outcome Measures	Main Findings
2004 Dolansky and Moore [69]	65 adults aged ≥70 years after CABG; two-group longitudinal comparative study	Supervised phase II CR involving cycling, treadmill walking, and risk-factor education	Dynamic and static balance; gait	CR participation was associated with better dynamic balance and gait; adjusted static-balance differences were not significant.
2012 Busch et al. [70]	173 adults aged ≥75 years after CABG; randomised controlled trial	Usual inpatient CR with or without additional resistance and progressive balance training	6MWT; TUG	Additional training produced greater improvements in 6MWT and TUG.
2014 Yamamoto et al. [71]	92 adults aged ≥65 years with IHD; retrospective cohort study	Conventional CR with or without 5-min standing balance training	One-leg standing; postural stability index; walking speed	Postural stability and walking speed improved more; one-leg standing showed no between-group difference.
2017 Rengo et al. [67]	196 adults aged ≥65 years with mixed cardiac diagnoses; prospective cohort study	Supervised phase II aerobic and resistance CR	SPPB and its components	SPPB improved clinically meaningfully in those with low baseline function; gains reflected gait speed and chair-rise, not balance.
2019 Segev et al. [72]	26 older adults with mixed CVD; exploratory randomised controlled trial	Traditional CR with or without 20-min stability and coordination training replacing the warm-up	TUG; BESS; FR	TUG and BESS improved more, whereas FR did not; BESS gains partially declined by 4 weeks.
2020 McGuire et al. [73]	33 community-dwelling adults aged ≥65 years with NYHA class II–III HF; pilot randomised wait-list-controlled trial	Weekly supervised balance and resistance training plus home exercise	DGI; TUG; 30-s STS; ABC; mCTSIB	DGI improved more at 12 weeks but declined during follow-up; other outcomes showed no group differences.
2021 Beigienė et al. [11]	63 adults aged ≥65 years after PCI or CABG for ACS; pilot randomised controlled trial	Conventional inpatient CR alone or with traditional- or device-based resistance and balance training	SPPB; 6MWT	SPPB and 6MWT improved across groups, with no added benefit from complementary training.
2021 Kitzman et al. [74]	349 adults aged 60–99 years with ADHF; multicentre randomised attention-controlled trial	Tailored progressive strength, balance, mobility, and endurance rehabilitation with home maintenance	SPPB and its components; 6MWT	Total SPPB, all components including standing balance, and 6MWT improved more with rehabilitation.
2021 Nakaya et al. [75]	75 adults aged ≥60 years with ADHF; randomised controlled trial	Standard CR with or without balance and resistance training and early interval cycling	SPPB and its components; gait speed; TUG; single-leg standing; 6MWT	Total SPPB, gait speed, and chair-rise improved more; the SPPB balance component, TUG, single-leg standing, and 6MWT showed no between-group differences at the end of intervention.
2021 Tamulevičiūtė-Prascienė et al. [76]	116 adults aged ≥65 years after valve surgery or intervention; randomised controlled trial	Inpatient CR with or without additional resistance and balance training, followed by home exercise	SPPB; 5MWT; 6MWT	Both groups improved similarly; SPPB-defined frailty was lower in the intervention group at 3 months, but follow-up data were incomplete.
2022 Brosved et al. [77]	26 adults aged ≥80 years after ACS; randomised controlled trial	Hospital-based aerobic, resistance, and balance training, plus home-based aerobic and resistance exercise	SPPB; TUG; one-leg standing; ABC; 6MWT	6MWT, total SPPB, one-leg standing, and balance confidence improved more; TUG and the SPPB balance component showed no between-group differences.
2024 Hirashiki et al. [20]	90 adults aged ≥65 years with worsening CVD; randomised controlled trial	Cycle ergometer exercise plus conventional resistance or robot-assisted video-game balance training	SPPB; TUG; 10-m gait speed; FIM motor	SPPB, TUG, and gait speed improved similarly; FIM motor improved more with robot-assisted training.

ABC: Activities-specific Balance Confidence Scale; ACS: acute coronary syndrome; ADHF: acute decompensated heart failure; BESS: Balance Error Scoring System; CABG: coronary artery bypass grafting; CR: cardiac rehabilitation; CVD: cardiovascular disease; DGI: Dynamic Gait Index; FIM: Functional Independence Measure; FR: functional reach; HF: heart failure; IHD: ischaemic heart disease; mCTSIB: modified Clinical Test of Sensory Interaction on Balance; NYHA: New York Heart Association; PCI: percutaneous coronary intervention; SPPB: Short Physical Performance Battery; TUG: Timed Up and Go test; 5MWT: 5-m walk test; 6MWT: 6-min walk test; 30-s STS: 30-s sit-to-stand test.

## Data Availability

No new data were created or analyzed in this study. Data sharing is not applicable to this article.

## Supplementary Materials

The following supporting information can be downloaded at: https://www.mdpi.com/article/10.3390/jcm15176657/s1, File S1: Living Strong Exercise Programme.

## References

[1] (2025). Global Burden of Cardiovascular Diseases and Risks 2023 Collaborators. Global, Regional, and National Burden of Cardiovascular Diseases and Risk Factors in 204 Countries and Territories, 1990–2023. J. Am. Coll. Cardiol..

[2] Alfaraidhy M.A., Regan C., Forman D.E. (2022). Cardiac rehabilitation for older adults: Current evidence and future potential. Expert Rev. Cardiovasc. Ther..

[3] Wang Y., Wang X., Wang C., Zhou J. (2024). Global, Regional, and National Burden of Cardiovascular Disease, 1990–2021: Results from the 2021 Global Burden of Disease Study. Cureus.

[4] Rich M.W., Chyun D.A., Skolnick A.H., Alexander K.P., Forman D.E., Kitzman D.W., Maurer M.S., McClurken J.B., Resnick B.M., Shen W.K. (2016). Knowledge Gaps in Cardiovascular Care of the Older Adult Population: A Scientific Statement from the American Heart Association, American College of Cardiology, and American Geriatrics Society. J. Am. Coll. Cardiol..

[5] Qu C., Liao S., Zhang J., Cao H., Zhang H., Zhang N., Yan L., Cui G., Luo P., Zhang Q. (2024). Burden of cardiovascular disease among elderly: Based on the Global Burden of Disease Study 2019. Eur. Heart J. Qual. Care Clin. Outcomes.

[6] Aïdoud A., Gana W., Poitau F., Debacq C., Leroy V., Nkodo J.A., Poupin P., Angoulvant D., Fougère B. (2023). High Prevalence of Geriatric Conditions Among Older Adults with Cardiovascular Disease. J. Am. Heart Assoc..

[7] Beigienė A., Petruševičienė D., Barasaitė V., Kubilius R., Macijauskienė J. (2021). Frailty and Different Exercise Interventions to Improve Gait Speed in Older Adults after Acute Coronary Syndrome. Medicina.

[8] Steinmetz C., Krause L., Sulejmanovic S., Kaumkötter S., Hartog J., Scheenstra B., Flohr S., Mengden T., Grefe C., Knoglinger E. (2024). Evaluation of frailty in geriatric patients undergoing cardiac rehabilitation after cardiac procedure: Results of a prospective, cross-sectional study. BMC Sports Sci. Med. Rehabil..

[9] Forman D.E., Maurer M.S., Boyd C., Brindis R., Salive M.E., Horne F.M., Bell S.P., Fulmer T., Reuben D.B., Zieman S. (2018). Multimorbidity in Older Adults with Cardiovascular Disease. J. Am. Coll. Cardiol..

[10] Esser R., Mondragon A., Larbaneix M., Esteban M., Farges C., Nisse Durgeat S., Maurou O., Harboun M. (2026). Core Competencies of the Modern Geriatric Cardiologist: A Framework for Comprehensive Cardiovascular Care in Older Adults. J. Clin. Med..

[11] Beigienė A., Petruševičienė D., Barasaitė V., Kubilius R., Macijauskienė J. (2021). Cardiac rehabilitation and complementary physical training in elderly patients after acute coronary syndrome: A pilot study. Medicina.

[12] O’Neill D., Forman D.E. (2020). The importance of physical function as a clinical outcome: Assessment and enhancement. Clin. Cardiol..

[13] Mori E., Aoyagi Y., Kono Y., Asai H., Tomita H., Izawa H. (2023). Exploring the factors associated with decreased dynamic balance ability in older patients with heart failure. Heart Lung.

[14] Forman D.E., Rich M.W., Alexander K.P., Zieman S., Maurer M.S., Najjar S.S., Cleveland J.C., Krumholz H.M., Wenger N.K. (2011). Cardiac care for older adults. Time for a new paradigm. J. Am. Coll. Cardiol..

[15] Kim D.H., Rich M.W. (2016). Patient-Centred Care of Older Adults with Cardiovascular Disease and Multiple Chronic Conditions. Can. J. Cardiol..

[16] Silveira H., Lima J., Plácido J., Ferreira J.V., Ferreira R., Laks J., Deslandes A. (2023). Dual-Task Performance, Balance and Aerobic Capacity as Predictors of Falls in Older Adults with Cardiovascular Disease: A Comparative Study. Behav. Sci..

[17] Raisi A., Piva T., Myers J., Zerbini V., Menegatti E., Lembo M., Michelon S., Meneghini I., Grazzi G., Mazzoni G. (2025). Increased Walking Speed Reduces Hospitalization Rates in Patients with Cardiovascular Disease During Exercise-Based Secondary Prevention. J. Clin. Med..

[18] Jansen J., Marshall P.W., Benatar J.R., Cross R., Lindbom T.K., Kingsley M. (2024). Low-Intensity Resistance Exercise in Cardiac Rehabilitation: A Narrative Review of Mechanistic Evidence and Clinical Implications. J. Clin. Med..

[19] Lutz A.H., Forman D.E. (2022). Cardiac rehabilitation in older adults: Apropos yet significantly underutilized. Prog. Cardiovasc. Dis..

[20] Hirashiki A., Shimizu A., Kamihara T., Kokubo M., Hashimoto K., Ueda I., Sato K., Kawamura K., Itoh N., Murohara T. (2024). Randomized Controlled Trial of Cardiac Rehabilitation Using the Balance Exercise Assist Robot in Older Adults with Cardiovascular Disease. J. Cardiovasc. Dev. Dis..

[21] Baethge C., Goldbeck-Wood S., Mertens S. (2019). SANRA-a scale for the quality assessment of narrative review articles. Res. Integr. Peer Rev..

[22] Damluji A.A., Forman D.E., Wang T.Y., Chikwe J., Kunadian V., Rich M.W., Young B.A., Page R.L., DeVon H.A., Alexander K.P. (2023). Management of Acute Coronary Syndrome in the Older Adult Population: A Scientific Statement from the American Heart Association. Circulation.

[23] Forman D.E., Kuchel G.A., Newman J.C., Kirkland J.L., Volpi E., Taffet G.E., Barzilai N., Pandey A., Kitzman D.W., Libby P. (2023). Impact of Geroscience on Therapeutic Strategies for Older Adults with Cardiovascular Disease: JACC Scientific Statement. J. Am. Coll. Cardiol..

[24] Eschalier R., Massoullié G., Boirie Y., Blanquet M., Mulliez A., Tartière P.L., Anker S., D’Agrosa Boiteux M.C., Richard R., Jean F. (2021). Sarcopenia in patients after an episode of acute decompensated heart failure: An underdiagnosed problem with serious impact. Clin. Nutr..

[25] Gary R. (2012). Evaluation of frailty in older adults with cardiovascular disease: Incorporating physical performance measures. J. Cardiovasc. Nurs..

[26] Pulignano G., Del Sindaco D., Di Lenarda A., Alunni G., Senni M., Tarantini L., Cioffi G., Tinti M.D., Barbati G., Minardi G. (2016). Incremental Value of Gait Speed in Predicting Prognosis of Older Adults with Heart Failure: Insights from the IMAGE-HF Study. JACC Heart Fail..

[27] Studenski S., Perera S., Patel K., Rosano C., Faulkner K., Inzitari M., Brach J., Chandler J., Cawthon P., Barrett-Connor E. (2011). Gait Speed and Survival in Older Adults. JAMA.

[28] Kamiya K., Hamazaki N., Matsue Y., Mezzani A., Corrà U., Matsuzawa R., Nozaki K., Tanaka S., Maekawa E., Noda C. (2018). Gait speed has comparable prognostic capability to six-minute walk distance in older patients with cardiovascular disease. Eur. J. Prev. Cardiol..

[29] Ozawa T., Yamashita M., Seino S., Kamiya K., Kagiyama N., Konishi M., Saito H., Saito K., Ogasahara Y., Maekawa E. (2021). Standardized gait speed ratio in elderly patients with heart failure. ESC Heart Fail..

[30] Tanaka S., Kamiya K., Hamazaki N., Matsuzawa R., Nozaki K., Nakamura T., Yamashita M., Maekawa E., Noda C., Yamaoka-Tojo M. (2019). Short-Term Change in Gait Speed and Clinical Outcomes in Older Patients with Acute Heart Failure. Circ. J..

[31] Uchida S., Kamiya K., Hamazaki N., Nozaki K., Ichikawa T., Nakamura T., Yamashita M., Maekawa E., Reed J.L., Yamaoka-Tojo M. (2021). Prognostic utility of dynapenia in patients with cardiovascular disease. Clin. Nutr..

[32] Nakamura T., Kamiya K., Hamazaki N., Matsuzawa R., Nozaki K., Ichikawa T., Yamashita M., Maekawa E., Reed J.L., Noda C. (2021). Quadriceps Strength and Mortality in Older Patients with Heart Failure. Can. J. Cardiol..

[33] Fried L.P., Tangen C.M., Walston J., Newman A.B., Hirsch C., Gottdiener J., Seeman T., Tracy R., Kop W.J., Burke G. (2001). Frailty in Older Adults: Evidence for a Phenotype. J. Gerontol. A Biol. Sci. Med. Sci..

[34] Pavasini R., Serenelli M., Celis-Morales C.A., Gray S.R., Izawa K.P., Watanabe S., Colin-Ramirez E., Castillo-Martínez L., Izumiya Y., Hanatani S. (2019). Grip strength predicts cardiac adverse events in patients with cardiac disorders: An individual patient pooled meta-analysis. Heart.

[35] Ozcan E.B., Saglam M., Vardar-Yagli N., Calik-Kutukcu E., Inal-Ince D., Altinsoy M., Kaya E.B. (2022). Impaired Balance and Gait Characteristics in Patients with Chronic Heart Failure. Heart Lung Circ..

[36] Pollock A.S., Durward B.R., Rowe P.J., Paul J.P. (2000). What is balance?. Clin. Rehabil..

[37] Horak F.B. (2006). Postural orientation and equilibrium: What do we need to know about neural control of balance to prevent falls?. Age Ageing.

[38] Teloudi A., Anifanti M., Chatzinikolaou K., Grouios G., Hatzitaki V., Chouvarda I., Kouidi E. (2024). Assessing Static Balance, Balance Confidence, and Fall Rate in Patients with Heart Failure and Preserved Ejection Fraction: A Comprehensive Analysis. Sensors.

[39] Del Buono M.G., Arena R., Borlaug B.A., Carbone S., Canada J.M., Kirkman D.L., Garten R., Rodriguez-Miguelez P., Guazzi M., Lavie C.J. (2019). Exercise Intolerance in Patients with Heart Failure: JACC State-of-the-Art Review. J. Am. Coll. Cardiol..

[40] Fedorowski A., Ricci F., Hamrefors V., Sandau K.E., Chung T.H., Muldowney J.A.S., Gopinathannair R., Olshansky B. (2022). Orthostatic Hypotension: Management of a Complex, But Common, Medical Problem. Circ. Arrhythm. Electrophysiol..

[41] Sturnieks D.L., St George R., Lord S.R. (2008). Balance disorders in the elderly. Neurophysiol. Clin..

[42] Sibley K.M., Beauchamp M.K., Van Ooteghem K., Straus S.E., Jaglal S.B. (2015). Using the systems framework for postural control to analyze the components of balance evaluated in standardized balance measures: A scoping review. Arch. Phys. Med. Rehabil..

[43] Franchignoni F., Horak F., Godi M., Nardone A., Giordano A. (2010). Using psychometric techniques to improve the Balance Evaluation Systems Test: The mini-BESTest. J. Rehabil. Med..

[44] Herman T., Giladi N., Hausdorff J.M. (2011). Properties of the ’timed up and go’ test: More than meets the eye. Gerontology.

[45] Shumway-Cook A., Brauer S., Woollacott M. (2000). Predicting the Probability for Falls in Community-Dwelling Older Adults Using the Timed Up & Go Test. Phys. Ther..

[46] Mancini M., Horak F.B. (2010). The relevance of clinical balance assessment tools to differentiate balance deficits. Eur. J. Phys. Rehabil. Med..

[47] Dobberke J., Baritello O., Hadzic M., Voller H., Eichler S., Salzwedel A. (2022). Test-retest reliability of center of pressure measures for postural control assessment in older cardiac patients. Gait Posture.

[48] Aflalo J., Truong C., Nicolai A., Gouzer L., Morisset B., Bertin-Hugault F., Ricard D., Quijoux F. (2026). Impact of sensory afferences in postural control quantified by force platform in healthy older adults: Systematic review and meta-analysis. Exp. Gerontol..

[49] Olsen S., Rashid U., Allerby C., Brown E., Leyser M., McDonnell G., Alder G., Barbado D., Shaikh N., Lord S. (2023). Smartphone-based gait and balance accelerometry is sensitive to age and correlates with clinical and kinematic data. Gait Posture.

[50] Olsen S., Rashid U., Barbado D., Suresh P., Alder G., Khan Niazi I., Taylor D. (2024). The validity of smartphone-based spatiotemporal gait measurements during walking with and without head turns: Comparison with the GAITRite(R) system. J. Biomech..

[51] Rashid U., Barbado D., Olsen S., Alder G., Elvira J.L.L., Lord S., Niazi I.K., Taylor D. (2022). Validity and Reliability of a Smartphone App for Gait and Balance Assessment. Sensors.

[52] Prisco G., Pisani N., Romano M., Amato F., Esposito F., Donisi L. (2026). Validity of Wearable Inertial Sensors for Postural Sway Analysis: A Systematic Review. Diagnostics.

[53] Muehlbauer T., Gollhofer A., Granacher U. (2015). Associations Between Measures of Balance and Lower-Extremity Muscle Strength/Power in Healthy Individuals Across the Lifespan: A Systematic Review and Meta-Analysis. Sports Med..

[54] Kinugawa S., Takada S., Matsushima S., Okita K., Tsutsui H. (2015). Skeletal Muscle Abnormalities in Heart Failure. Int. Heart J..

[55] Helbostad J.L., Sturnieks D.L., Menant J., Delbaere K., Lord S.R., Pijnappels M. (2010). Consequences of lower extremity and trunk muscle fatigue on balance and functional tasks in older people: A systematic literature review. BMC Geriatr..

[56] Pasma J.H., Engelhart D., Maier A.B., Schouten A.C., van der Kooij H., Meskers C.G. (2015). Changes in sensory reweighting of proprioceptive information during standing balance with age and disease. J. Neurophysiol..

[57] Peterka R.J. (2018). Sensory integration for human balance control. Handb. Clin. Neurol..

[58] Doumas M., Krampe R.T. (2010). Adaptation and reintegration of proprioceptive information in young and older adults’ postural control. J. Neurophysiol..

[59] Henry M., Baudry S. (2019). Age-related changes in leg proprioception: Implications for postural control. J. Neurophysiol..

[60] Petrigna L., Gentile A., Mani D., Pajaujiene S., Zanotto T., Thomas E., Paoli A., Palma A., Bianco A. (2021). Dual-Task Conditions on Static Postural Control in Older Adults: A Systematic Review and Meta-Analysis. J. Aging Phys. Act..

[61] Woollacott M., Shumway-Cook A. (2002). Attention and the control of posture and gait: A review of an emerging area of research. Gait Posture.

[62] Li K.Z.H., Bherer L., Mirelman A., Maidan I., Hausdorff J.M. (2018). Cognitive Involvement in Balance, Gait and Dual-Tasking in Aging: A Focused Review from a Neuroscience of Aging Perspective. Front. Neurol..

[63] Al-Yahya E., Dawes H., Smith L., Dennis A., Howells K., Cockburn J. (2011). Cognitive motor interference while walking: A systematic review and meta-analysis. Neurosci. Biobehav. Rev..

[64] Freeman R., Wieling W., Axelrod F.B., Benditt D.G., Benarroch E., Biaggioni I., Cheshire W.P., Chelimsky T., Cortelli P., Gibbons C.H. (2011). Consensus statement on the definition of orthostatic hypotension, neurally mediated syncope and the postural tachycardia syndrome. Auton. Neurosci..

[65] Bourke R., Doody P., Pérez S., Moloney D., Lipsitz L.A., Kenny R.A. (2024). Cardiovascular Disorders and Falls Among Older Adults: A Systematic Review and Meta-Analysis. J. Gerontol. A Biol. Sci. Med. Sci..

[66] Cersosimo A., Longo Elia R., Condello F., Colombo F., Pierucci N., Arabia G., Matteucci A., Metra M., Adamo M., Vizzardi E. (2026). Cardiac rehabilitation in patients with atrial fibrillation. Minerva Cardiol. Angiol..

[67] Rengo J.L., Savage P.D., Shaw J.C., Ades P.A. (2017). Directly Measured Physical Function in Cardiac Rehabilitation. J. Cardiopulm. Rehabil. Prev..

[68] Forman D.E., Arena R., Boxer R., Dolansky M.A., Eng J.J., Fleg J.L., Haykowsky M., Jahangir A., Kaminsky L.A., Kitzman D.W. (2017). Prioritizing Functional Capacity as a Principal End Point for Therapies Oriented to Older Adults with Cardiovascular Disease: A Scientific Statement for Healthcare Professionals from the American Heart Association. Circulation.

[69] Dolansky M.A., Moore S.M. (2004). Effects of Cardiac Rehabilitation on the Recovery Outcomes of Older Adults After Coronary Artery Bypass Surgery. J. Cardiopulm. Rehabil..

[70] Busch J.C., Lillou D., Wittig G., Bartsch P., Willemsen D., Oldridge N., Bjarnason-Wehrens B. (2012). Resistance and balance training improves functional capacity in very old participants attending cardiac rehabilitation after coronary bypass surgery. J. Am. Geriatr. Soc..

[71] Yamamoto S., Matsunaga A., Wang G., Hoshi K., Kamiya K., Noda C., Kimura M., Yamaoka-Tojo M., Masuda T. (2014). Effect of Balance Training on Walking Speed and Cardiac Events in Elderly Patients with Ischemic Heart Disease. Int. Heart J..

[72] Segev D., Hellerstein D., Carasso R., Dunsky A. (2019). The effect of a stability and coordination training programme on balance in older adults with cardiovascular disease: A randomised exploratory study. Eur. J. Cardiovasc. Nurs..

[73] McGuire R., Honaker J., Pozehl B., Hertzog M. (2020). BASIC Training: A Pilot Study of Balance/Strengthening Exercises in Heart Failure. Rehabil. Nurs..

[74] Kitzman D.W., Whellan D.J., Duncan P., Pastva A.M., Mentz R.J., Reeves G.R., Nelson M.B., Chen H., Upadhya B., Reed S.D. (2021). Physical Rehabilitation for Older Patients Hospitalized for Heart Failure. N. Engl. J. Med..

[75] Nakaya Y., Akamatsu M., Ogimoto A., Kitaoka H. (2021). Early cardiac rehabilitation for acute decompensated heart failure safely improves physical function (PEARL study): A randomized controlled trial. Eur. J. Phys. Rehabil. Med..

[76] Tamulevičiūtė-Prascienė E., Beigienė A., Thompson M.J., Balnė K., Kubilius R., Bjarnason-Wehrens B. (2021). The impact of additional resistance and balance training in exercise-based cardiac rehabilitation in older patients after valve surgery or intervention: Randomized control trial. BMC Geriatr..

[77] Brosved M., Hirlekar G., Philip Wigh J., Sundberg H., Zidén L., Karlsson T., Albertsson P., Bäck M. (2022). Effects of Cardiac Rehabilitation on Physical Fitness, Physical Function, and Self-reported Outcomes in Patients ≥ 80 yr: A Randomized Controlled Trial. J. Cardiopulm. Rehabil. Prev..

[78] Dajime P.F., Smith H., Zhang Y. (2022). Bespoke Exergames for Balance Improvement and Fall Risk Reduction in Community-Dwelling Older Adults: A Systematic Review and Meta-Analysis of Randomized Controlled Trials. IEEE Trans. Games.

[79] Lee J., Phu S., Lord S.R., Okubo Y. (2024). Effects of immersive virtual reality training on balance, gait and mobility in older adults: A systematic review and meta-analysis. Gait Posture.

[80] Zak M., Sikorski T., Michalska A., Sztandera P., Szczepanowska-Wolowiec B., Brola W., Courteix D., Dutheil F. (2024). The Effects of Physiotherapy Programmes, Aided by Virtual Reality Solutions, on Balance in Older Women: A Randomised Controlled Trial. J. Clin. Med..

[81] Jiao Y., Dajime P.F., Zhang Q., Reading S., Smith M.-C., Zhang Y. (2025). A clinically oriented VR-based treadmill training system for post-stroke rehabilitation: Integration of motor learning and control principles. Virtual Real..

[82] de Medeiros C.S.P., Farias L.B.A., Santana M.C.D.L., Pacheco T.B.F., Dantas R.R., da Costa Cavalcanti F.A. (2024). A systematic review of exergame usability as home-based balance training tool for older adults usability of exergames as home-based balance training. PLoS ONE.

[83] Tuena C., Pedroli E., Trimarchi P.D., Gallucci A., Chiappini M., Goulene K., Gaggioli A., Riva G., Lattanzio F., Giunco F. (2020). Usability Issues of Clinical and Research Applications of Virtual Reality in Older People: A Systematic Review. Front. Hum. Neurosci..

[84] Blasco-Peris C., Alcolea Garrido J.P., Seguí B., Zaragoza R., Climent-Paya V., Fuertes-Kenneally L., Manresa-Rocamora A., Sanz-Rocher A., Baladzhaeva S., Sarabia J.M. (2025). Exergaming System for Exercise-Based Cardiac Rehabilitation in Patients with Heart Failure: Development and Usability Assessment Study of a Device Prototype. JMIR Serious Games.

[85] Xie Q., Zhang Q., Zhang Y., Sheng B., Wang X., Luo J., Huang J. (2025). Research on upper limb motor function evaluation and rehabilitation training system of stroke patients based on artificial intelligence: A usability and feasibility study from therapist and patient perspectives. Digit. Health.

[86] Cheng Q., Li F., Zhang Q., Yu H., Wang S. (2025). The Impact of Virtual Reality on Cardiopulmonary Function and Adherence in Cardiac Rehabilitation Patients: A Systematic Review and Meta-Analysis. Healthcare.

[87] Flint K.M., Stevens-Lapsley J., Forman D.E. (2020). Cardiac Rehabilitation in Frail Older Adults with Cardiovascular Disease: A New Diagnostic and Treatment Paradigm. J. Cardiopulm. Rehabil. Prev..

[88] Terbraak M., Major M., Jørstad H., Scholte Op Reimer W., van der Schaaf M. (2024). Home-based cardiac rehabilitation in older adults: Expert-recommendations for physiotherapist-led care to improve daily physical functioning and reduce comorbidity-related barriers. Eur. J. Physiother..

[89] Tinetti M.E., Naik A.D., Dindo L., Costello D.M., Esterson J., Geda M., Rosen J., Hernandez-Bigos K., Smith C.D., Ouellet G.M. (2019). Association of Patient Priorities-Aligned Decision-Making with Patient Outcomes and Ambulatory Health Care Burden Among Older Adults with Multiple Chronic Conditions: A Nonrandomized Clinical Trial. JAMA Intern. Med..

[90] Slone S.E., D’Aoust R., Wright R.S., Yandle V.A., Barringhaus K.G. (2026). Virtual and Remote Cardiac Rehabilitation for Older Adults: Current Evidence and Implementation Strategies. Curr. Geriatr. Rep..

[91] Thomas R.J., Beatty A.L., Beckie T.M., Brewer L.C., Brown T.M., Forman D.E., Franklin B.A., Keteyian S.J., Kitzman D.W., Regensteiner J.G. (2019). Home-Based Cardiac Rehabilitation: A Scientific Statement from the American Association of Cardiovascular and Pulmonary Rehabilitation, the American Heart Association, and the American College of Cardiology. J. Am. Coll. Cardiol..

[92] Lynn M.H., Luo G., Tomasi M., Pundlik S., Houston K.E. (2020). Measuring Virtual Reality Headset Resolution and Field of View: Implications for Vision Care Applications. Optom. Vis. Sci..

[93] Almajid R., Tucker C., Keshner E., Vasudevan E., Wright W.G. (2021). Effects of wearing a head-mounted display during a standard clinical test of dynamic balance. Gait Posture.

[94] Akiduki H., Nishiike S., Watanabe H., Matsuoka K., Kubo T., Takeda N. (2003). Visual-vestibular conflict induced by virtual reality in humans. Neurosci. Lett..

[95] Rmadi H., Maillot P., Artico R., Baudouin E., Hanneton S., Dietrich G., Duron E. (2023). Tolerance of immersive head-mounted virtual reality among older nursing home residents. Front. Public Health.

[96] Cieślik B., Serweta A., Federico S., Szczepańska-Gieracha J. (2021). Altered postural stability in elderly women following a single session of head-mounted display virtual reality. Acta Bioeng. Biomech..

[97] Thomas S., Mackintosh S., Halbert J. (2010). Does the ‘Otago exercise programme’ reduce mortality and falls in older adults?: A systematic review and meta-analysis. Age Ageing.

[98] Dent E., Morley J.E., Cruz-Jentoft A.J., Woodhouse L., Rodríguez-Mañas L., Fried L.P., Woo J., Aprahamian I., Sanford A., Lundy J. (2019). Physical Frailty: ICFSR International Clinical Practice Guidelines for Identification and Management. J. Nutr. Health Aging.

[99] Hashimoto K., Hirashiki A., Ozaki K., Kawamura K., Sugioka J., Tanioku S., Sato K., Ueda I., Itoh N., Nomoto K. (2022). Benefits of a Balance Exercise Assist Robot in the Cardiac Rehabilitation of Older Adults with Cardiovascular Disease: A Preliminary Study. J. Cardiovasc. Dev. Dis..

