# Supplementary material for: Balance and Mobility Impairment in Older Adults with Cardiovascular Disease Before and After Rehabilitation: A Narrative Review
Source: J Clin Med. 2026 Aug 28;15(17):6657. doi: 10.3390/jcm15176657 (PMC13566091; doi:10.3390/jcm15176657)
Supplement: Supplementary file 1 [file jcm-15-06657-s001.zip › jcm-4498594-supplementary.pdf]

LIVING STRONG

Exercise Programme

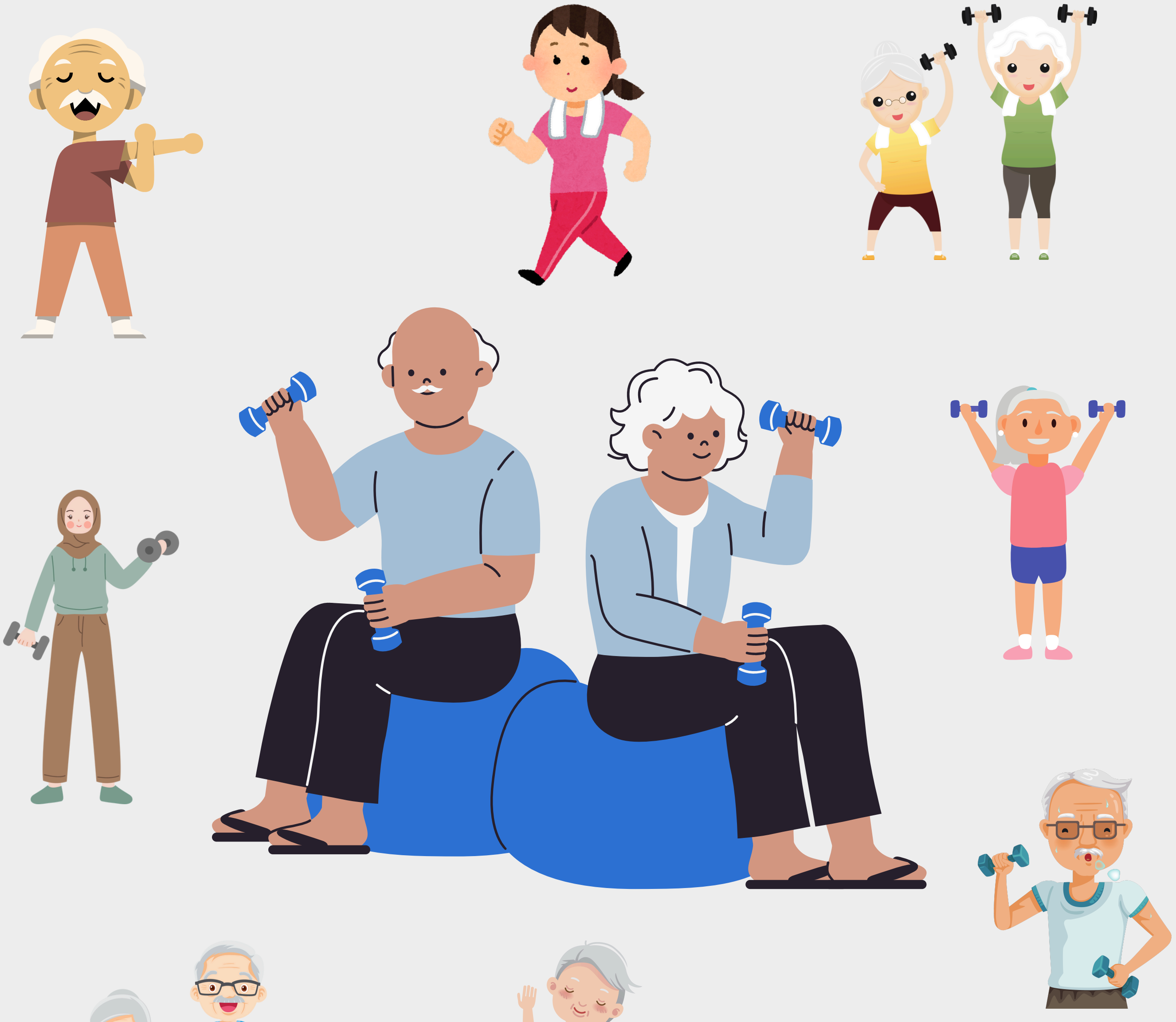

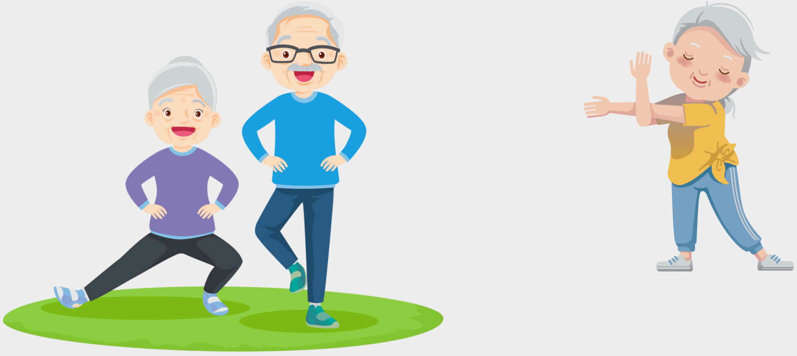

Sasha Douglas

Accredited Exercise Physiologist

Clinical Researcher

Te Whatu Ora
Health New Zealand
Te Toka Tumai Auckland

THE UNIVERSITY OF AUCKLAND
Te Whare Wānanga o Tāmaki Makaurau
NEW ZEALAND

EXERCISE PROGRAMME

To remember to do these exercises, it can be helpful to pair the habit with something you already do. Have a think... what is a habit you do every morning without fail…? Brushing your teeth! Use brushing your teeth as a reminder to start these exercises next.

Others find that in the morning they feel most motivated, have less duties or other engagements and can put time into themselves ☺ It helps having a plan to help overcome any obstacles that may come up. Try to plan your sessions into your week in a way that works for you. See below for an example of how your week could look.

SAMPLE WEEK

MONDAY	TUESDAY	WEDNESDAY	THURSDAY	FRIDAY
Mobility + Balance	Mobility + Balance	Mobility + Balance	Mobility + Balance	Mobility + Balance
Lower Body Resistance		Upper Body Resistance		Lower Body Resistance

MOBILITY

HOW OFTEN DO I DO THIS?

Complete this 5x per week. It's designed to help reduce stiffness and keep your joints moving so you can perform your activities of everyday life

HOW MANY OF EACH?

2 sets of 12 repetitions

Complete each exercise 12 times, take a 60s break, then complete one more round

HOW SHOULD IT FEEL?

Think about a scale from 1-10 (with 1 feeling very easy, and 10 feeling like maximal effort), complete the repetitions so it feels like a 2-4 out of 10. They shouldn't feel strenuous

BREATHING

It is important to breathe normally throughout each exercise. Do not hold your breath

MOBILITY

Start here!

1

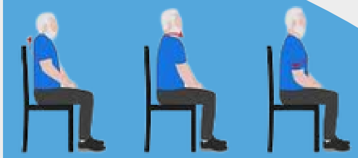

Lift shoulders up, rotate in a circle forwards and lower. Repeat in opposite direction

SHOULDER ROLLS

2

SEATED CAT/COW

Hands on knees. Arch back and open through chest/shoulders. Then round back and look downwards

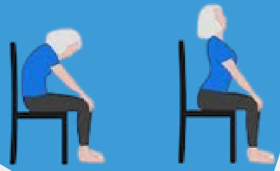

3

UPPER BODY ROTATION

Sit tall, arms crossed over chest. Rotate to one side, head looking over shoulder

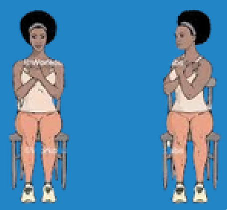

4

SIDE BEND

Reach arm up and over, feel a stretch down side of torso

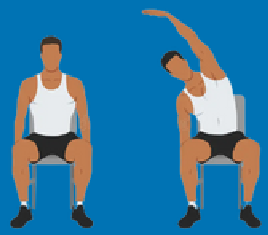

5

SEATED HINGES

Sit tall, keep a straight spine and hinge forwards. Don't let shoulders/spine round forwards

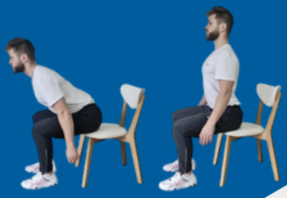

6

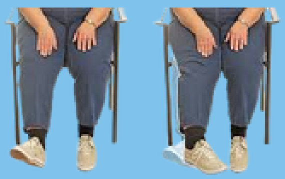

Create circles one foot at a time in one direction and repeat the other way

ANKLE ROLLS

BALANCE

HOW OFTEN DO I DO THIS?

Complete this 5x per week. It's designed to help increase your balance and confidence to perform activities in your everyday life

HOW MANY OF EACH?

2-3x 30-45s

Start by performing the exercise for 2 rounds of 30s on each leg (if applicable). Build up to 45s and 3 rounds.

SUPPORT/SAFETY

For each balance exercise, always be next to something you can lean on/hold onto for support. Start each exercise holding on to the support and let go once you feel steady

BREATHING

It is important to breathe normally throughout each exercise. Do not hold your breath

EXTRA TIP...

Brush and balance! As you are brushing your teeth, practice standing one foot in front of the other, or on one foot even (right next to the bench for support if needed)

BALANCE

Start here!

1 FEET TOGETHER BALANCE

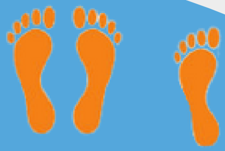

Stand tall with feet together, hold onto surface for support and remove hand when steady. Move to single foot balance when ready

2 TANDEM STANCE BALANCE

One foot in front of the other (or slightly to the side), hold on for support and remove hand when steady

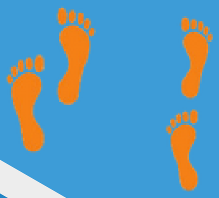

3 STANDING MARCHES

Sit/stand tall. Lift one leg keeping it bent, lower and swap legs. Don't move upper body

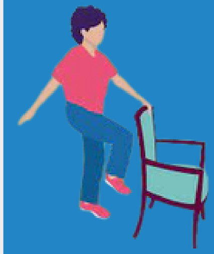

4 TANDEM WALKING

Place one foot directly in front of the other one at a time, moving forwards. Repeat backwards for an extra challenge

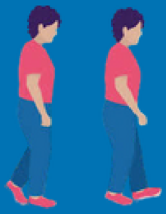

5 SIDEWAYS WALKING

Take small steps sideways. Have a slight bend in your knees for an extra challenge

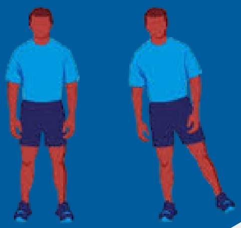

6 FIGURE 8'S

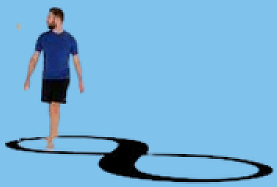

Walk in a figure 8, around an object one way, and around the other in the opposite direction

LOWER BODY

HOW OFTEN DO I DO THIS?

Complete this 5x per week. It's designed to help reduce stiffness and keep your joints moving so you can perform your activities of everyday life

HOW MANY OF EACH?

2-3 sets of 8-12 repetitions

Start by performing an exercise 8 times. Rest for 60s, then repeat another 8 times. Once that starts to feel 'easier', increase it to 10 or 12 repetitions. Once that feels 'easier', add another set on. You can add ankle weights for extra resistance

HOW SHOULD IT FEEL?

Think about a scale from 1-10 (with 1 feeling very easy, and 10 feeling like maximal effort), complete the repetitions so it feels like a ~4 out of 10. You'll know when to increase the sets and repetitions when your intensity rating gets lower

BREATHING

Breathe normally throughout each exercise. Do not hold your breath

LOWER BODY

Start here!

1 SEATED LEG EXTENSIONS

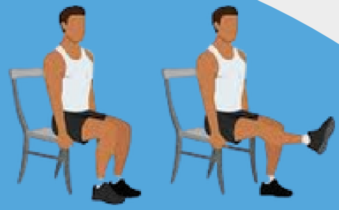

Extend one leg out straight and squeeze top of thigh. Don't move upper body

2 SEATED TOWEL SQUEEZES

Place a rolled-up towel between your knees and squeeze inner thighs together

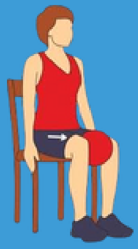

3 SIDE LEG LIFTS

Keep foot flexed and leg straight as you carry it out to the side. Squeeze side of your hip to lift leg

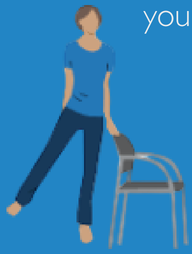

4 HAMSTRING CURLS

Squeeze hamstring as you lift one foot up off the ground behind you. Keep knees next to each other

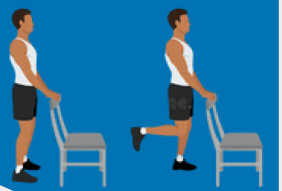

5 CALF RAISES

Hold onto support, keep body in line as you lift heels off ground. Don't let hips swing forwards

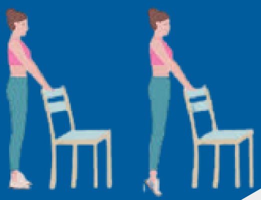

6 SIT TO STAND

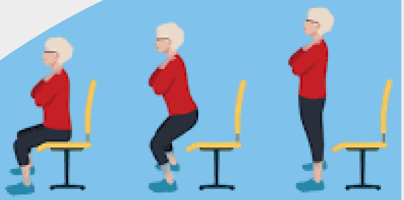

Feet shoulder width apart, knees in line with toes and chest up. Stand up and slowly lower back down

UPPER BODY

HOW OFTEN DO I DO THIS?

Complete this 5x per week. It's designed to help reduce stiffness and keep your joints moving so you can perform your activities of everyday life

HOW MANY OF EACH?

2-3 sets of 8-12 repetitions

Start by performing an exercise 8 times. Rest for 60s, then repeat another 8 times. Once that starts to feel 'easier', increase it to 10 or 12 repetitions. Once that feels 'easier', add another set on. You can add ankle weights for extra resistance

HOW SHOULD IT FEEL?

Think about a scale from 1-10 (with 1 feeling very easy, and 10 feeling like maximal effort), complete the repetitions so it feels like a ~4 out of 10. You'll know when to increase the sets and repetitions when your intensity rating gets lower

BREATHING

Breathe normally throughout each exercise. Do not hold your breath

UPPER BODY

Start here!

1 ARM GOALPOSTS

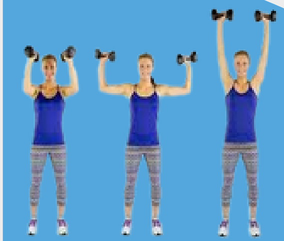

Weights (or tinned cans in hands), arms in front at 90 degrees, open out to side, push up overhead. Lower and repeat

2 BANDED PULL BACK

Wrap band around pole, squeeze your back as you row arms back, keep elbows tucked in

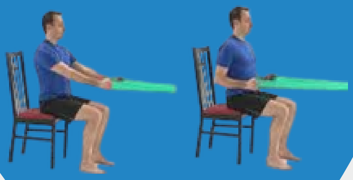

3 WALL PUSH UP

Hands at shoulder height on wall, bend elbows and lower towards wall. Keep elbows tucked into sides

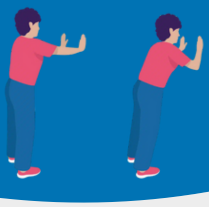

4 TRICEP KICKBACK

Lean forwards, elbows tucked into sides, extend arms straight and squeeze triceps

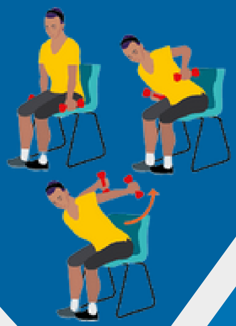

5 BICEP CURLS

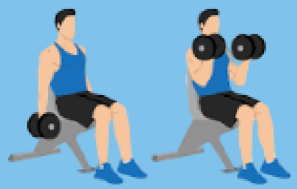

Hold light weights (or tinned can), keep elbows at side of body as you squeeze your biceps to curl weights up
